# Properties and Structure of Deposited Nanocrystalline Coatings in Relation to Selected Construction Materials Resistant to Abrasive Wear

**DOI:** 10.3390/ma11071184

**Published:** 2018-07-10

**Authors:** Jacek Górka, Artur Czupryński, Marcin Żuk, Marcin Adamiak, Adam Kopyść

**Affiliations:** 1 Department of Welding Engineering, Silesian University of Technology, Konarskiego 18A, 44-100 Gliwice, Poland; artur.czuprynski@polsl.pl (A.C.); marcin.zuk@polsl.pl (M.Ż.); 2 Institute of Engineering Materials and Biomaterials, Silesian University of Technology, Konarskiego 18A, 44-100 Gliwice, Poland; marcin.adamiak@polsl.pl (M.A.); adam.kopysc@gmail.com (A.K.)

**Keywords:** abrasive wear, nanocrystalline layers, abrasion resistant plate, deposit weld

## Abstract

Presented in this work are the properties and structure characteristics of MMA (Manual Metal Arc) deposited nanocrystalline coatings (Fe-Cr-Nb-B) applied to an iron nanoalloy matrix on an S355N steel substrate in relation to selected construction materials resistant to abrasive wear currently used in industry. The obtained overlay welds were subjected to macro and microscopic metallographic examinations; grain size was determined by X-ray diffraction (XRD), and chemical composition of precipitates was determined by energy-dispersive X-ray spectroscopy (EDS) during scanning electron microscopy (SEM). The size of the crystalline grains of the Fe-Cr-Nb-B nanocrystalline microstructure was analyzed using an Xpert PRO X-ray diffractometer. Analysis of the test results of the obtained layers of arc-welded Fe-Cr-Nb-B-type alloy confirmed that the obtained layers are made of crystallites with a size of 20 nm, which classifies these layers as nanocrystalline. The obtained nanocrystalline coatings were assessed by hardness and with the use of metal-mineral abrasion testing. The results of the coatings’ properties tests were compared to HARDOX 400 alloy steel.

## 1. Introduction

Wearing of machine parts poses an important problem in terms of scientific, technical and economic potential. In most cases, the mechanisms of wear are highly complex and include many interrelated factors whose impact depends on the environment and working conditions of the part in question. The variety of the types of wear modes leads to the specialization of materials used in the construction of parts subject to abrasive wear in order to ensure the highest resistance to wearing of surface layers under specific operating conditions. One of the types of such specialized materials is Abrasion Resistant (AR) plates [1,2,3,4,5,6,7]. The structure and properties of the surface layer are, to a large extent, the main factor when considering materials for machine parts in terms of durability. In recent years, there has been dynamic research in the development of new abrasion-resistant materials containing layers of unique properties and structures differing significantly from previous work. In particular, new approaches such as improved hardness, resistance to impact loads and low coefficients of friction are the main concerns when developing new materials [8,9,10,11,12,13,14,15,16,17,18,19]. The rapid development of nano-structurally modified materials is foreshadowing an increase in their application in novel welding technologies. The wide variety of properties inherent to nano-structually modified materials are a paradigm shift, bringing new possibilities through the use of nanomaterial-based surfacing technologies. Nanomaterials are classified as single or multiphase polycrystals characterized by a grain size on the order of 1 × 10^−9^ m to 250 × 10^−9^ m in diameter. At the upper limit of this range, the term “very fine” is used with respect to grain size on the order of 250–1000 nm in diameter [20,21,22,23,24,25,26,27]. Nanocrystalline materials are structurally characterized by a high volume fraction at the grain boundaries, which significantly changes their physical, chemical and mechanical properties in comparison to conventional coarse grains, whose grain size is usually on the order of 10–300 µm. Until now, existing nanomaterials used for nanostructure coatings and layers have shown a significantly higher (many times) wear resistance compared to traditional steel alloy-based materials. Due to the high cost and continuous development of their production technology, nanostructural materials have not been widely applied [28,29,30,31,32,33].

## 2. Experimental Section

The purpose of the performed experiments was to compare the structure and properties of Fe-Cr-Nb-B nanocrystalline surface layers deposited by manual metal arc (MMA) welding with a covered electrode 3.2 mm in diameter to previously used abrasion-resistant materials. HARDOX 400 steel was used as a reference material in the assessment of resistance to metal-mineral abrasion. The following materials were tested in the experiments:

(1) Nanocrystalline layer deposited with a covered electrode—NANO (Fe-Cr-Nb-B)
(2) Abrasion-resistant plate—ABRECOPLATE
(3) Abrasion-resistant plate—CDP
(4) Abrasion-resistant layer deposited by covered electrode—ABRADUR 64
(5) Abrasion-resistant layer deposited by GMA with a ceramo-metallic wire containing 50% WC (tungsten carbide)
(6) Abrasion-resistant sheet—HARDOX 400.

Metallographic examinations of the deposited coatings were carried out on a Zeiss SteREO Discovery, and LEICA MEF4A optical microscope (Leica Microsystems, Cambridge, UK) in addition to a Zeiss Supra 35 (SEM) scanning electron microscope (Carl Zeiss GmbH, Jena, Germany). Metallographic images were taken of transverse sections with respect to weld deposition direction. The specimens were prepared by standard metallographic techniques and etched in Nital solution. Phase compositions of the investigated materials in addition to the size of the crystallographic grains of nanocrystalline microstructure were determined using an Xpert PRO X-ray diffractometer with step data logging, employing the filtered K@ X-rays. The chemical composition of precipitates was determined by energy-dispersive X-ray spectroscopy (EDS) with an EDAX detector. Hardness tests were conducted on a Zwick Roell ZHR hardness tester based on the Rockwell method, while cross-sectional hardness was measured using the Vickers method with a Future-Tech FM-700 tester (Future-Tech Corp., Kawasaki, Kanagawa Prefecture, Japan).

### 2.1. Nanocrystalline Layer Deposited with Covered Fe-Cr-Nb-B Electrode

**Figure 1:**
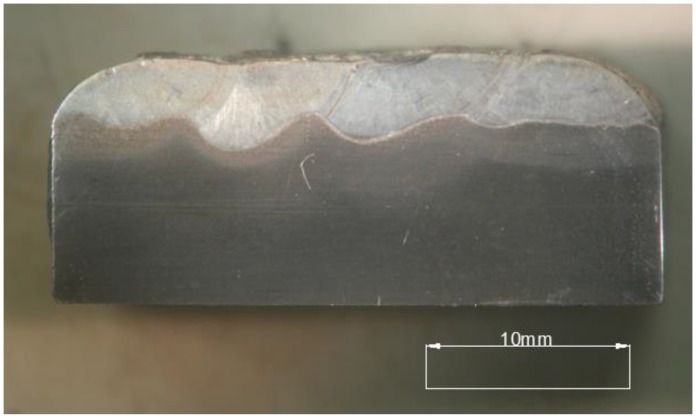
Macrostructure of the cross-section of a nanocrystalline layer, the average width of the overlay layer is 3 mm.

The Fe-Cr-Nb-B nanocrystalline layer, shown in Table 1, deposited by MMA with a 3.2 mm covered electrode on an S355N steel alloy substrate is shown in Figure 1. MMA deposit welding was performed with a constant direct current of 100 A in the flat position (PA). During welding, the electrode was set at an angle of 90° with respect to the surface of the welding base. The sheet surface was ground and pre-heated with a gas torch to a temperature of 80 °C.

According to the manufacturer′s data, the Fe-Cr-Nb-B nanocrystalline alloy electrodes mostly consist of very hard boron carbide fractions evenly distributed in a semi-amorphous iron alloy matrix, yielding a hardness of 67–70 HRC. The deposit weld should have a high abrasion resistance and an increased resistance to dynamic loads due to these characteristics. The electrodes can be used for both direct- and alternating-current welding.

### 2.2. Abrasion Resistant Plate—ABRECOPLATE

ABRECOPLATE abrasion-resistant materials are produced in the following formats: plates (straight or beveled, by special order), bars, buttons (in the shape of a dome, octagonal, protecting screws). ABRECOPLATE is a layered material composed of chromium-molybdenum white cast iron, metallurgically connected to a soft-structural steel underlying plate, as detailed in Table 2.

**Figure 2:**
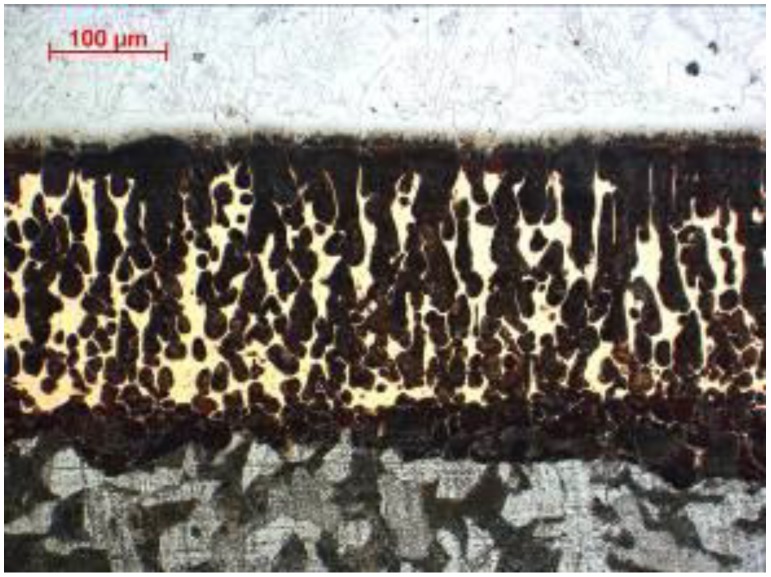
Microstructure of the intermediate layer of ABRECOPLATE cross-section.

ABRECOPLATE’s high abrasion-resistance properties are due to the structure of the surface layer. The special heat treatment (hardening throughout) of cast iron allows for the obtainment of a microstructure consisting of chromium-molybdenum carbides in an almost completely martensitic matrix. The substrate of these abrasive plates is a soft-structural steel. Undercoated cast iron is joined by soldering with a soft copper-based binder which ensures adequate stress transfer, shown in Figure 2.

An important advantage of ABRECOPLATE abrasive discs is the content of abrasive material in relation to the primer, which is 3:1.

### 2.3. CDP Plate

**Figure 3:**
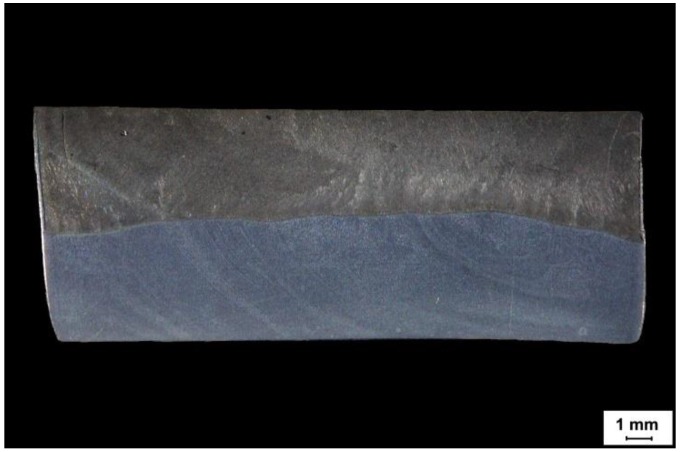
Macrostructure of a cross-section of CDP plate.

Abrasion-resistant plates are manufactured by welding a sheet of non-alloy, low-alloy or high-alloy steel with shielded powder wire or self-shielded wire, as shown in Table 3 and Figure 3.

The deposited layer exhibits a very high abrasion resistance and has a standard thickness of 3–18 mm. Typical dimensions of abrasion plates are: 1000 × 2000 mm, 1500 × 3000 mm, and 2000 × 3000 mm. It is possible to cut flat elements of any shape from abrasion plates and further shape them by bending and rolling. They are joined to the regenerated substrate with fillet welds, continuous or intermittent, depending on the type of abrasive plate load. The high content of carbon, chromium, and niobium allows for the obtainment of a structure similar to that of cast iron with the inclusion of very hard chromium borides, niobium carbides, and iron carbides.

### 2.4. Abrasion Resistant Layer Deposited by Covered ABRADUR 64 Electrode

**Figure 4:**
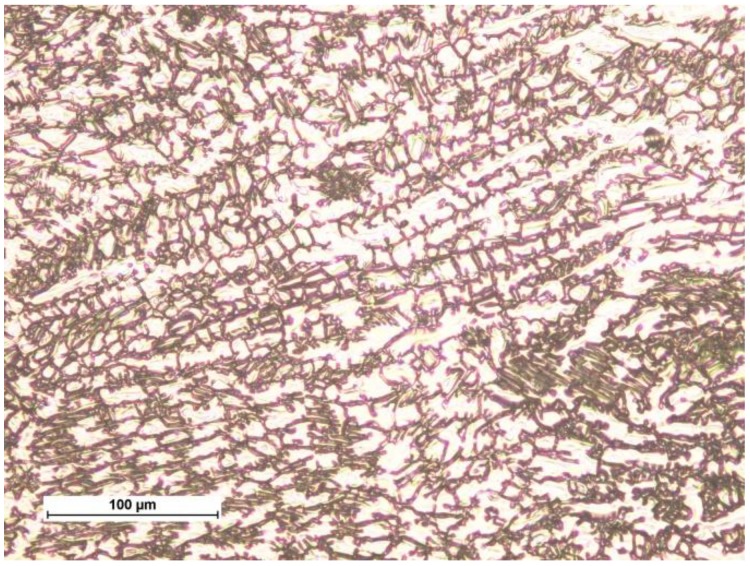
Microstructure of austenitic intermediate layer.

The abrasion-resistant layer was deposited by manual welding of an S335JR steel with a covered electrode, DIN 8555: E 10-UM-65-GR with a diameter of 5.0 mm and a welding current of 270 A. The welding process was carried out using a buffer layer made with a covered electrode, ERWS 19-12-3 L with a diameter of 3.25 mm and a welding current of 110 A, as shown in Figure 4. The purpose of the buffer layer was to transfer the stresses between the base material and hardfacing. The chemical composition and properties of the weld metal, ABRADUR 64 are given in Table 4.

### 2.5. Abrasion Resistant Layer Formed by GMA with Ceramo-Metallic Wire

The abrasion-resistant layer was made by single-layer GMA welding of 15 HM steel using a ceramo-metallic wire with a 50% nickel carbide content of tungsten carbide (WC). The analysis of the chemical composition is shown in Table 5.

### 2.6. HARDOX 400 Steel Sheet

HARDOX type steels are defined as “high-quality abrasion-resistant steels”. This group of materials is derived from low-alloy steels destined for heat treatment and belongs to a new generation of machinable and weldable structural steels. Materials made of HARDOX steel are used where resistance to abrasion is required such as in the presence of variable loads, e.g., feeders, crushers, sieves, shafts, elements of incline lift, conveyors, blades, gears and chains, dumpers, loaders, trucks, motor-carriages, dozers, loading buckets, and screw conveyors. All types of HARDOX steel are delivered in the hardened condition (water-hardened). In the case of specifically required hardnesses, tempering is also performed. These steels can be bent, cut, drilled, machined, or turned under strictly defined conditions. HARDOX sheets can be machined using high-speed steel (HSS) or tools made of sintered carbides. The chemical composition and properties of HARDOX 400 steel are given in Table 6.

## 3. Results and Discussion

### 3.1. ASTM G65-00 Metal–Mineral Abrasion Resistance Test

**Figure 5:**
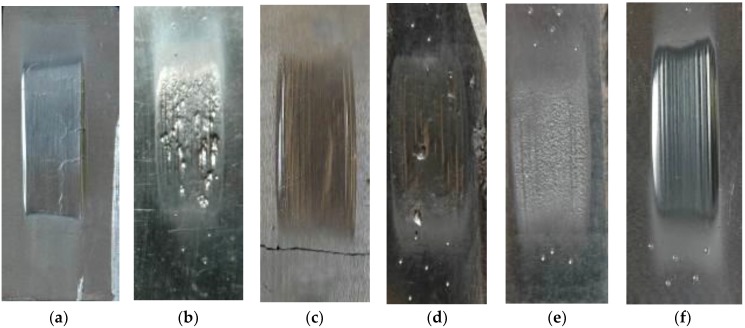
Abrasion tests samples following the abrasion test: (**a**) NANO; (**b**) ABRECOPLATE; (**c**) CDP; (**d**) ABRADUR 64; (**e**) WC; (**f**) HARDOX 400.

**Figure 6:**
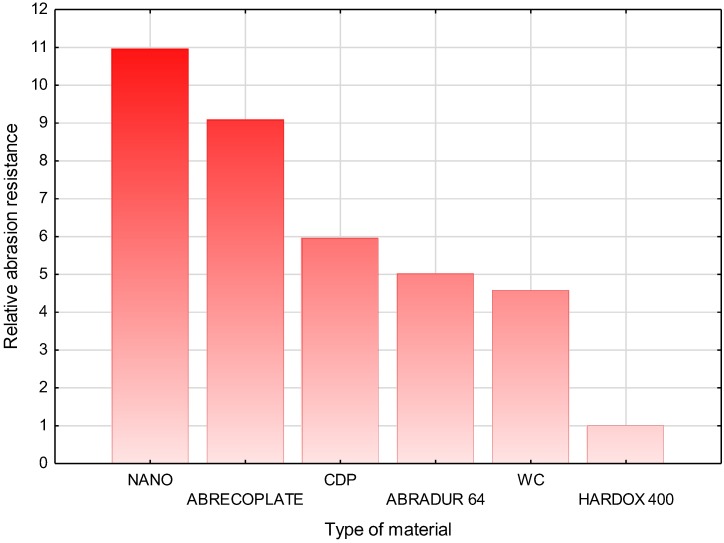
Comparison of the results of relative abrasive wear resistance of selected construction materials.

The tests of abrasive wear resistance for the selected materials were carried out in accordance with ASTM G 65-00. Procedure A, which is the most demanding examination of abrasion resistance, was used for the tests. During the test, the sample was mounted in a special fixture in which it was clamped to a rubber wheel 228.6 mm in diameter. The test sample was pressed against the rubber wheel with a force of 130 N. Abrasive in the form of granular sand (gradation 250 µm) was delivered through the nozzle at the location of sample contact with the rubber wheel. The abrasive flow rate was 300–400 g/min. The wheel rotated in the direction corresponding to the abrasive flow, at a speed of 200 rpm through 6000 revolutions. The tested samples were 25 × 75 × thickness of the sample, mm, as shown in Figure 5. The mass loss was determined using an analytical balance precise to 0.0001 g (measurement accuracy up to 5 decimal places). To compare the results of abrasion resistance, the density of plates and abrasive layers was determined. As a measure of abrasion, the volume loss of the sample (mm^3^) was determined, and is shown in Equation (1), Table 7, and Figure 6.

(1) $$Volume\;loss\;\left({\mathrm{mm}}^{3}\right)=mass\;loss\;\left(g\right)\;:\;density\;\left(\frac{g}{{\mathrm{cm}}^{3}}\right)\times 1000$$

The tests have shown that the relative resistance to metal-to-metal abrasive wear of the nanocrystalline layer is 11 times higher when compared to the Hardox400 reference metal sheet.

### 3.2. Metallographic Testing

**Figure 7:**
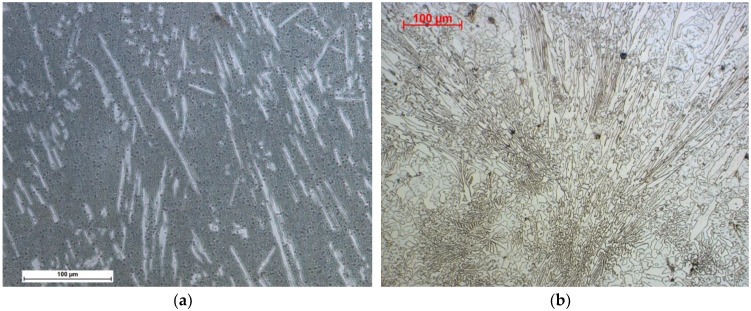

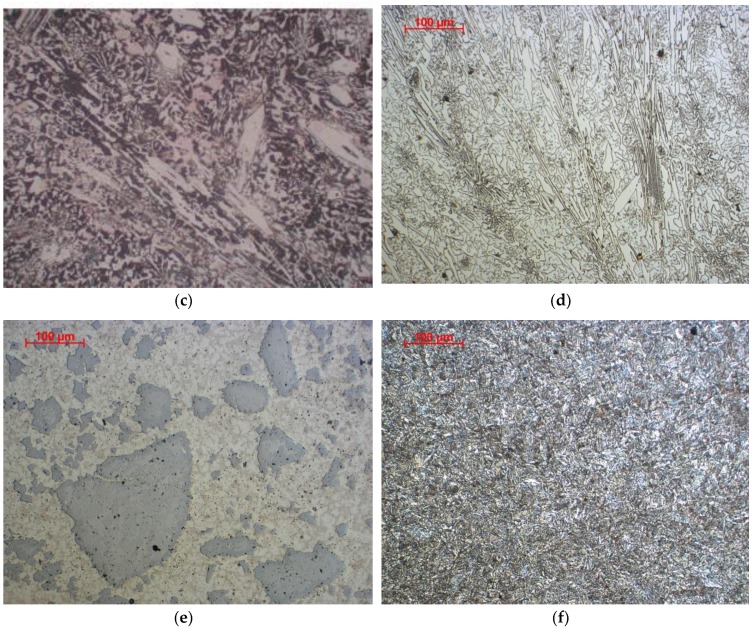
Microstructure of layers resistant to abrasive wear. (**a**) NANO; (**b**) ABRECOPLATE; (**c**) CDP; (**d**) ABRADUR 64; (**e**) WC; (**f**) HARDOX 400; Reagents selected for abrasion resistant material.

The microscopic observations made it possible to determine the microscopic structure of the materials studied. The observations, carried out using a light microscope, did not show internal defects in the layers due to welding methods and material defects in the case of HARDOX 400 sheet, ABRECOPLATE, and CDP plates, Figure 7.

**Figure 8:**
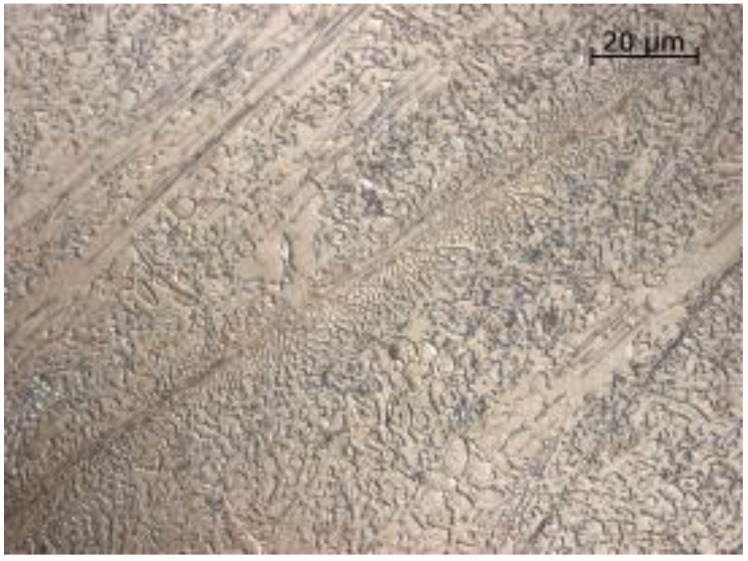
Microstructure of a nanocrystalline layer with eutectic like carbides of NANO (Fe-Cr-Nb-B).

**Figure 9:**
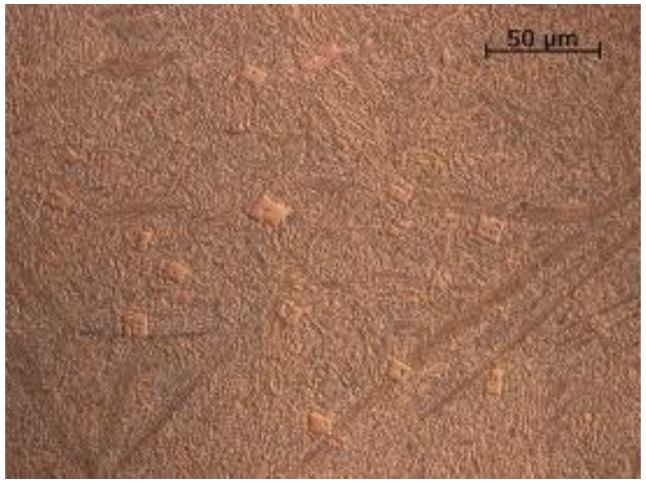
Microstructure of a nanocrystalline layer with carbide precipitates of NANO (Fe-Cr-Nb-B).

Metallographic examinations carried out on a scanning microscope technique (SE) revealed large amounts of primary carbide precipitates highly dispersed in the zone of the nanocrystalline matrix, as shown in Figure 8 and Figure 9.

**Figure 10:**
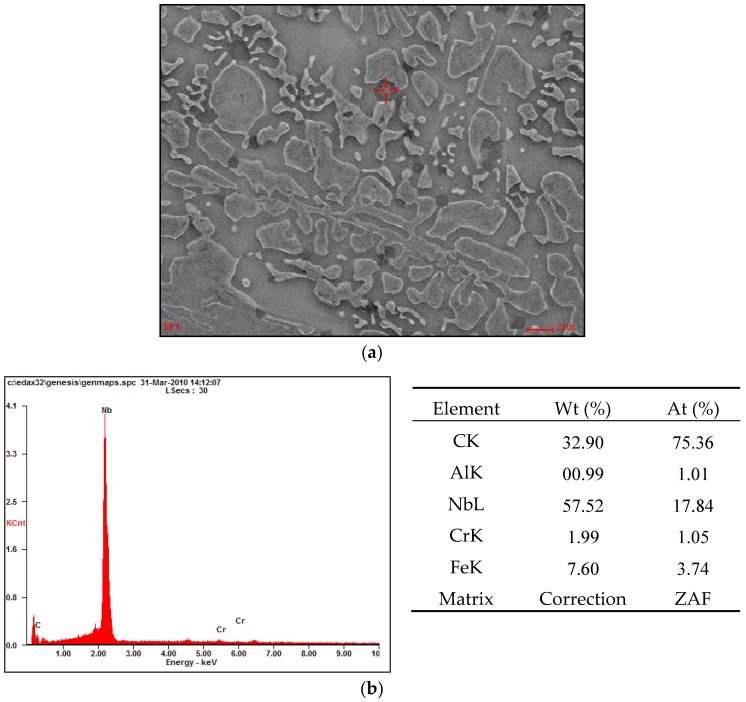
(**a**) SEM image of the nanostructural layered microstructure over the EDS analysis area—a precipitation of niobium carbide; (**b**) the EDS spectrum with the above indicated spot-separation of niobium carbide and results of the quantitative elemental analysis.

**Figure 11:**
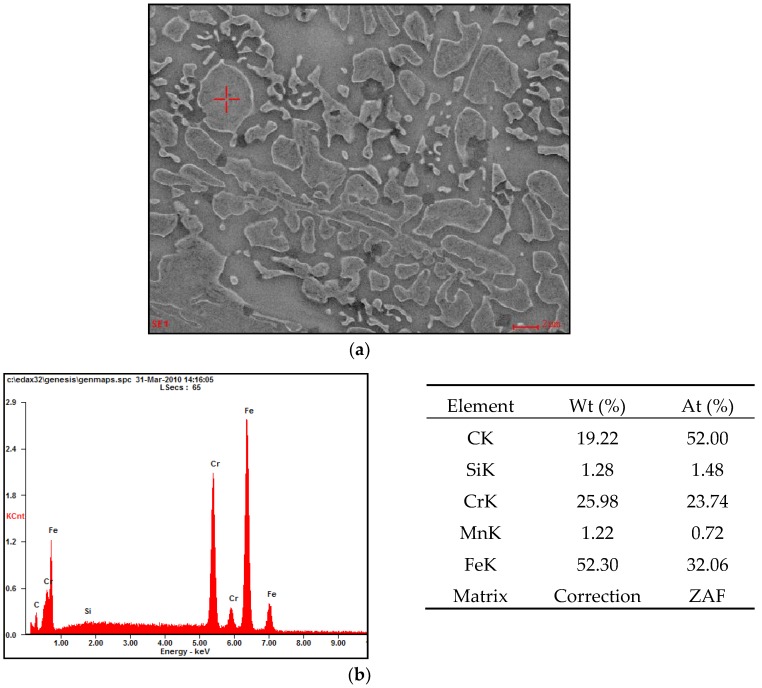
(**a**) SEM image of the nanostructural layer microstructure over the EDS analysis area—a precipitation of chromium carbide; (**b**) the EDS spectrum showing the above indicated spot-separation of chromium carbide and results of the quantitative elemental analysis.

SEM analysis showed that the observed carbide precipitates which appeared larger in size were niobium and chromium carbides, shown in Figure 10 and Figure 11.

The size of the crystallographic grains of the Fe-Cr-Nb-B nanocrystalline microstructure was measured using an Xpert PRO X-ray diffractometer by PANalytical and a computerized radiation recording system equipped with a cobalt lamp at 40 kV and 30 mA current with a strip detector, in the Bragg angle range of 30–120°. Based on calculations of crystalline sizes carried out using the Scherrer Equation (2), it was found that the average grain size of the layered microstructure measured in the direction perpendicular to the deposited substrate was approximately 20 nm.

(2) $$D=\frac{K\cdot \lambda }{B_{\mathrm{struct}}\cdot \cos\theta }$$

where: *D*—the average size of the crystallite in the direction perpendicular to the planes of deflection; *K*—Scherrer constant (0.98); *λ*—wavelength; *B_struct_*—width of reflexes; *θ*—the angle of reflection.

**Figure 12:**
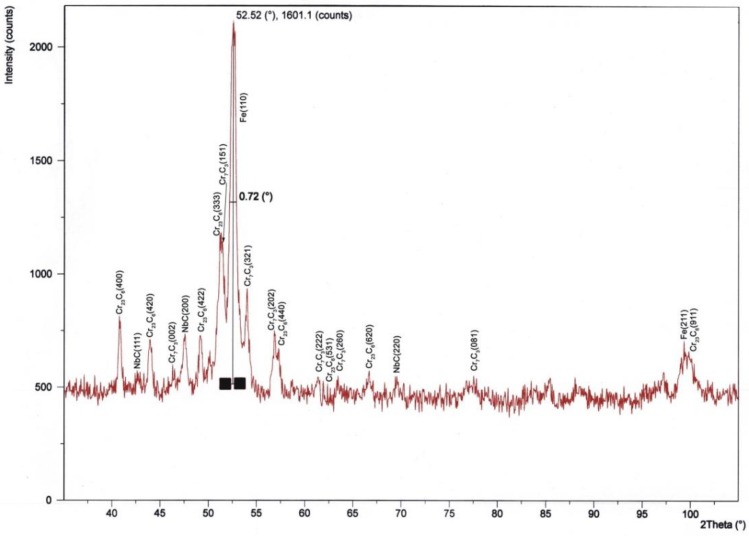
The diffraction pattern of the nanocrystalline Fe-Cr-Nb-B layer.

Analysis of the diffraction pattern of the nanocrystalline layers of Fe-Cr-Nb-B showed the presence of reflections from the three types of carbides (Figure 12):

- Cr_7_C_3_ from the lattice plane (002), (151), (321), (202), (222), (260), (081);
- Cr_23_C_6_ from the lattice plane (400), (420), (422), (333), (440), (531), (620), (911);
- NbC from the lattice plane (111), (200), (220).

### 3.3. Hardness Testing

**Figure 13:**
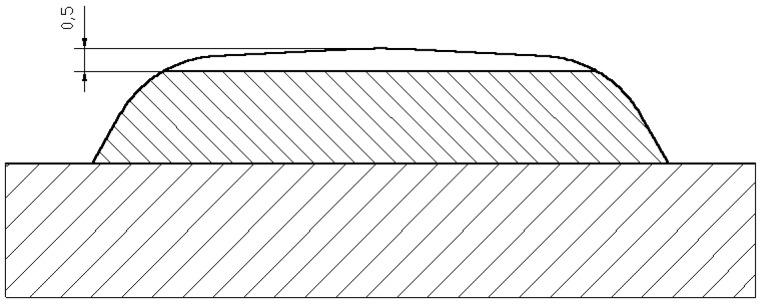
Preparation for hardness testing.

**Figure 14:**
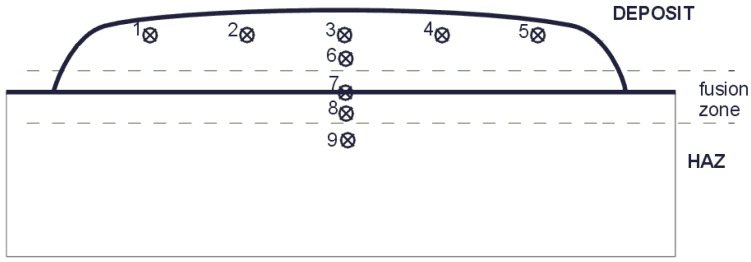
Hardness test area.

**Figure 15:**
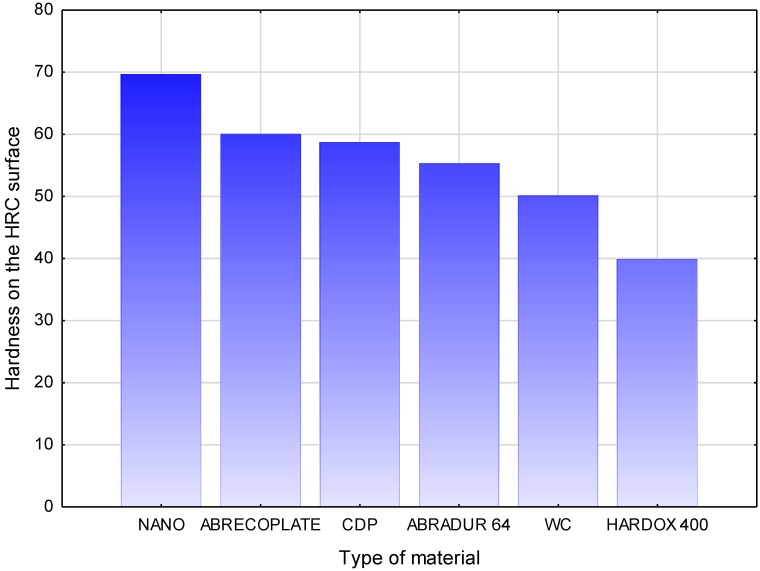
Average hardness of the surface of tested construction materials.

In order to determine the hardness of the tested materials, the Rockwell hardness measurement was carried out in five places on the weld face/sheet surface and in four locations on the deposit weld/sheet cross-section using the Vickers method at a load of 1000 g, shown in Figure 13 and Figure 14. Hardness measurement results are shown in Table 8 and Figure 15.

## 4. Conclusions

The metallographic examinations of the materials selected for the tests did not show any internal or external defects in the layers formed by MMA welding with coated electrodes and GMA welding. Analysis of the test results of the obtained layers of arc-welded Fe-Cr-Nb-B-type alloy confirmed:

- The obtained layers are made of crystallites with a size of 20 nm, which classifies these layers as nanocrystalline.
- Numerous carbide precipitates with empirical formula Cr_7_C_3_, Cr_23_C_6_, and NbC were observed and detected in the samples.

The ABRECOPLATE plate has a structure of white cast iron with chromium-molybdenum carbide precipitates. To join this plate with a low-carbon steel support plate, a soft binder was used on the copper matrix, which perfectly transfers the stresses occurring between the layers. In the case of CDP, the surface layer structure is a chromium cast iron containing many primary carbides.

The layer created with ABDADUR 64 coated electrodes possesses a eutectic iron structure with numerous precipitates of niobium and chromium carbides. The use of an austenitic steel buffer layer, in this case, allowed for crack avoidance that could propagate through to the substrate material. The layer formed by GMA surfacing with a ceramo-metallic wire is characterized by a nickel matrix containing many tungsten carbides. As a result of the thermal cycle, it can be observed that the WC carbides are partially dissolved, which may reduce the abrasion resistance of such layers.

HARDOX 400 is characterized by a tempered martensite structure. The hardness measurements carried out on the ground face of abrasion-resistant layers showed that all materials have a hardness similar to the hardness quoted by the manufacturers. The highest hardness on the surface is characterized by the nanocrystalline layer, with a hardness of 70 HRC. The tests of resistance to abrasive wear of the metal-mineral type, according to ASTM G 65-00, have shown that the best usable properties are characterized by a layer made of Fe-Cr-Nb-B alloy. The metal-mineral abrasion resistance of this material is 11 times higher than a typical HARDOX 400 abrasion-resistant sheet.

## Figures and Tables

**Table 1:** Chemical composition and hardness of the tested deposit weld.

Chemical Composition, wt %							HRC
C	Cr	B	Nb	Mn	Si	Fe	
1.4	15.2	4.0	3.4	0.4	0.4	remainder	68–70

**Table 2:** Chemical composition and physical characteristics of the abrasion-resistant layer of ABRECOPLATE.

Chemical Composition, wt %							
C	Cr	Mo	Mn	Si		Ni	Fe
2.8–3.6	14.0–18.0	2.3–3.5	0.5–1.5	1.0 max.		5.0 max.	remainder
**Mechanical Properties**							
**HRC**		**Heat Resistance (°C)**			**Creep Resistance (°C)**		
64		540			595		

**Table 3:** Chemical composition of the surface deposit weld.

Chemical Composition, wt %							HRC
C	Cr	B	Nb	Mn	Si	Fe	
5.2	22.0	1.8	7.0	0.4	0.4	remainder	57–62

**Table 4:** Chemical composition and properties of the abrasion-resistant layer of ABRADUR 64-covered electrodes.

Chemical Composition, wt %				HRC
C	Cr	Nb	Fe	
7.0	24.0	7.0	remainder	64

**Table 5:** Chemical composition of the layer formed by GMA (Gas Metal Active) with a ceramo-metallic wire layer.

Mass Percent of Elements in Abrasion Resistant, wt %					
Ni	C	Si	Cr	B	WC
Remainder	0.4	2.5	3.0	1.5	50

**Table 6:** Chemical composition and mechanical properties of HARDOX 400.

Chemical Composition, wt %					
C	Mn	Mo	Cr	Si	Ni
0.14–0.32	1.60	0.25–0.60	0.30–1.40	0.70	0.25–1.50
**Mechanical Properties**					
**HBW**		**Tensile Strength (MPa)**		**Yield Strength (MPa)**	
370–430		1250		1000	

**Table 7:** ASTM G65-00 abrasion resistance test results.

Material/Density (g/cm^3^)	Sample Number	Sample Weight before Test (g)	Sample Weight after Test (g)	Mass Loss (g)	Average Mass Loss (g)	Average Volume Loss (mm^3^)	Relative Abrasion Resistance *
NANO/8.78	1	102.9477	102.8393	0.1084	0.1113	12.6765	10.95
	2	101.7964	101.6821	0.1143			
ABRECOPLATE/7.5961	1	173.7335	173.6133	0.1202	0.11635	15.3170	9.07
	2	173.6714	173.5589	0.1125			
CDP/7.1724	1	128.6154	128.4378	0.1776	0.1697	23.3881	5.94
	2	128.9438	128.7821	0.1617			
ABRADUR 64/7.1544	1	136.2893	136.0933	0.1960	0.19825	27.7102	5.01
	2	139.6675	139.4670	0.2005			
WC/10.6808	1	179.6026	179.3009	0.3017	0.32360	30.2974	4.58
	2	181.8750	181.5295	0.3455			
HARDOX 400/7.7115	1	62.1029	61.0320	1.0709	1.0691	138.8705	1.00
	2	62.5591	61.4918	1.0673			

* Relative abrasion resistance compared to HARDOX 400.

**Table 8:** Hardness testing results on the face and cross-section of deposit welds and sheets.

Material	HRC						HV 1			
	1	2	3	4	5	Average	6	7	8	9
NANO	69	70	69	71	69	69.60	679	289	179	188
ABRECOPLATE	60	58	61	61	62	60.04	663	154	148	106
CDP	62	59	58	61	58	58.70	643	299	179	182
ABRADUR 64	57	56	57	54	52	55.20	556	174	304	290
WC	54	50	48	51	49	50.04	563	486	180	167
HARDOX 400	41	40	39	40	39	39.80	380	378	377	378
